# Compressive Behavior and Microstructural Characteristics of Iron Hollow Sphere Filled Aluminum Matrix Syntactic Foams

**DOI:** 10.3390/ma8115432

**Published:** 2015-11-23

**Authors:** Attila Szlancsik, Bálint Katona, Kornél Májlinger, Imre Norbert Orbulov

**Affiliations:** 1 Department of Materials Science and Engineering, Műegyetem rakpart 3, Budapest 1111, Hungary; szlana045@gmail.com (A.S.); katona@eik.bme.hu (B.K.); vmkornel@eik.bme.hu (K.M.); 2 MTA–BME Research Group for Composite Science and Technology, Műegyetem rakpart 3, Budapest 1111, Hungary

**Keywords:** metal matrix composites, cellular materials, metallic foams, syntactic foams, hollow sphere, mechanical characterization, compression, microstructure, electron back-scattered diffraction, energy dispersive spectroscopy

## Abstract

Iron hollow sphere filled aluminum matrix syntactic foams (AMSFs) were produced by low pressure, inert gas assisted infiltration. The microstructure of the produced AMSFs was investigated by light and electron microscopy, extended by energy dispersive X-ray spectroscopy and electron back-scattered diffraction. The investigations revealed almost perfect infiltration and a slight gradient in the grain size of the matrix. A very thin interface layer that ensures good bonding between the hollow spheres and the matrix was also observed. Compression tests were performed on cylindrical specimens to explore the characteristic mechanical properties of the AMSFs. Compared to other (conventional) metallic foams, the investigated AMSFs proved to have outstanding mechanical properties (yield strength, plateau strength, *etc.*) and energy absorbing capability.

## 1. Introduction

Metal matrix syntactic foams (MMSFs) are hollow inclusion reinforced metal matrix composites. The hollow inclusions are usually spherical and they are made from high strength materials (glass, ceramics or metals). Therefore they ensure certain reinforcement and due to the hollow inclusions, the composite has a definite and more or less regular foam structure. The most common matrix material of the MMSFs is some kind of aluminum alloy (aluminum matrix syntactic foams (AMSFs)), but recently MMSFs with Mg [1,2,3,4,5], Zn [6,7], Ti [8] or heavier (but cheaper) steel [9,10,11,12,13,14,15] matrices were developed. In addition, steel based MMSFs were also investigated [16,17]. The hollow spheres are made from glass (amorphous SiO_2_ and other oxides), Al_2_O_3_, SiC [5,18] or less often some kind of Fe based (steel) alloy. Beside the reinforcing effect of the steel hollow spheres another important reason for their application is their relatively low cost compared to Al_2_O_3_ or SiC hollow spheres. This is an important economic issue in the spreading of MMSFs, therefore special efforts were made to apply other low cost filler materials [19,20,21,22,23]. Considering the production methods, stir casting [24] and gravitational casting [14,20,21] are the most commonly applied methods, however, successful tests proved the reliability of a powder metallurgy route too [8,25,26].

In our case, the reinforcement consists of a set of iron hollow spheres. The properties of the most similar MMSFs were investigated by the research team of Rabiei [27,28]. They developed a new closed cell foam type, called composite metal foams (CMFs). CMF is comprised of steel hollow spheres packed into a random dense arrangement, with the interstitial space between spheres infiltrated with a casting aluminum alloy (generally speaking, they can be considered as MMSFs too). The measured density of the composite material was 2.4 g·cm^−3^, with a relative density of 41.5%. The developed CMFs showed superior compressive strength (67 MPa over a region of 10%–50% strain) and energy absorption capacity. The densification began at ~50% strain, and the energy absorption up to 50% strain was ~30 MJ·m^−3^. Later, Neville and Rabiei [29,30] produced CMFs by powder metallurgy. CMFs were processed by filling the vacancies between densely packed steel hollow spheres with steel powder and sintering them into a solid cellular structure. The relative densities of the products were in the range of 32.4%–38.9%. Although denser than other foams, the produced CMFs displayed again superior compressive strengths and energy absorption capabilities. The plateau strength to density ratio for the carbon steel matrix samples were in the range of 12–31.9 MPa·g^−1^ cm^3^ and for stainless steel matrix samples 43.7 MPa·g^−1^ cm^3^. The energy absorption up to densification for carbon steel and stainless steel samples ranged from 18.9 to 41.7 MJ·m^−3^ and ~67.8 MJ·m^−3^, respectively. Subsequently, the same research group [17] characterized the compressive fatigue properties of the afore-mentioned CMFs. Under compression fatigue loading, the CMF samples proved high cyclic stability at maximum stress levels up to 90 MPa. The deformation of the CMF samples was divided into three stages: (i) linear increase in strain with fatigue cycles; (ii) minimal strain accumulation in large number of cycles; and (iii) rapid strain accumulation within few cycles resulting in complete failure. Considering the structure of the foams, CMFs underwent a uniform deformation, unlike the regular metal foams, which deformed along collapse bands at weaker sections. The most significant features that determine the fatigue life of the CMFs were considered to be the sphere wall thickness and diameter, sphere and matrix materials, processing techniques and the strength of bonding between the spheres and matrix. The performance of the CMFs under simple, three-point bending was also evaluated along with simultaneous acoustic emission monitoring [31]. The results showed high maximum bending strength up to 86 MPa. Acoustic emission behavior showed that the dominating failure mechanism of cast CMF was the brittle fracture of intermetallic phases that exist at the interface of the spheres, whereas in powder metallurgy samples, the failure was governed by the propagation of pre-existing micro-porosities in the matrix resulting in a complete ductile failure. More recently, Rabiei and Garcia-Avila [16,32] investigated the effect of loading rate (up to 26 m·s^−1^) on the mechanical properties of CMFs. The yield and plateau strength as well as the energy absorption capabilities of the CMFs were increased with increasing loading rate and by decreasing sphere sizes. The features controlling the life time and performance of CMFs under static and dynamic loading were categorized into two main groups: (i) controls the yield and plateau strength of the foam at lower strain levels, that includes bonding strength between the spheres and matrix (depending on the spheres’ surface roughness and on the chemical composition gradient in the interface layer); and (ii) controls the relative density, densification strain and plateau strength at higher strain levels (depending on the sphere diameter and the porosity content in both spheres and matrix).

The aim of this study is to explore the microstructure and the mechanical properties of Al alloy based, iron hollow sphere filled syntactic foams.

## 2. Results and Discussion

In this section the results of the microstructural and mechanical investigations are discussed. All tests were performed on Fe hollow sphere filled AMSFs produced by low-pressure infiltration. The investigated materials were designated by a code consisting of the matrix (the amounts of alloying elements are in mass percent, see Table 2) and the heat treatment, for example: AlSi12-O designates an AMSF specimen with AlSi12 matrix (contains 12 wt % Si) in solution treated condition (O), AlCu5-T6 is for an AMSF with AlCu5 matrix (contains 5 wt % Cu) and in T6 (solution treated and artificially aged) condition.

### 2.1. Microstructure

**Figure 1:**
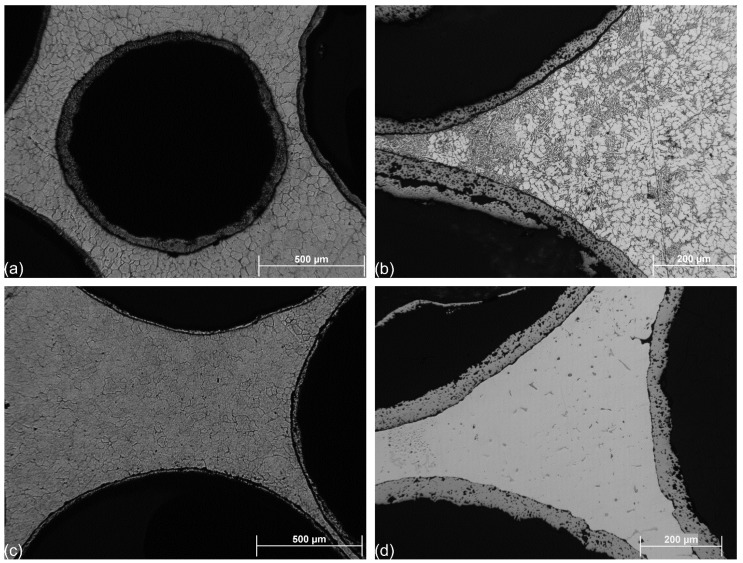
Micrograph of a typical GM grade iron hollow sphere in (**a**) Al99.5-O; (**b**) AlSi12-O; (**c**) AlMgSi1-O; and (**d**) AlCu5-O AMSF.

The typical and general microstructure of the investigated AMSFs is represented in Figure 1. This figure shows typical micrographs of the as-cast and etched (Keller’s reagent) samples. In Figure 1a, a hollow sphere surrounded by others is highlighted in the Al99.5-O sample. The applied GM (abbreviation for the trade name GloboMet) grade hollow spheres are spherical in shape and have quite high porosity in their wall. Note that the small gaps between the hollow spheres were completely filled by the pressure infiltration (for example in the right side of Figure 1b or right-bottom in Figure 1c). The grains of the matrix material are equiaxed with typical size of ~10–50 μm. In Figure 1b, the very fine Al-Si eutectic structure of AlSi12-O samples can be clearly observed. The density of the Si lamellae seems to be higher near to the hollow spheres. Figure 1c is similar to Figure 1a, and presents the microstructure of AlMgSi1-O sample, equiaxed grains and perfect infiltration can be seen. Figure 1d shows the AlCu5-O sample. The micrograph exhibits fine structure and some primer CuAl_2_ precipitations.

From a microstructural point of view, one of the most critical parts of the particle reinforced composites is the interface layer between the reinforcement (GM grade hollow spheres in this case) and the matrix material. This layer is responsible for the transfer of the loading from the matrix material to the reinforcing hollow spheres. In the Al-Fe system, a few chemical reactions (Equations (1)–(3)) can occur during the production process and in the liquid state of the infiltrating matrix material. The below listed reactions are diffusion reactions and the propelling force is the Fe concentration mismatch between the hollow spheres’ walls and the matrix material (note, that the last reaction occurs only in the case of AlSi12 and AlMgSi1 matrices).

(1) $$Fe_{solid}+Al_{liquid}\Leftrightarrow FeAl_{liquid\;solution}$$

(2) $$Fe_{solid}+3Al_{liquid}\Leftrightarrow Al_{3}Fe_{solid}$$

(3) $$Fe_{solid}+Si_{liquid}\Leftrightarrow FeSi_{solid}$$

**Figure 2:**
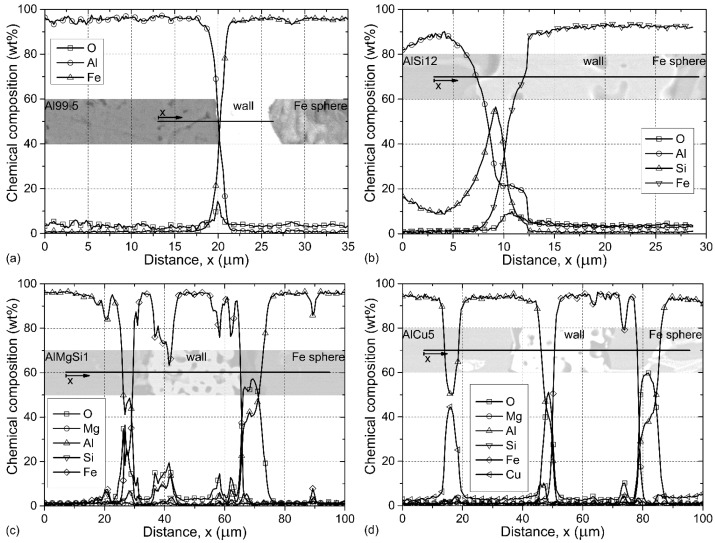
Line EDS profiles of (**a**) Al99.5-O; (**b**) AlSi12-O; (**c**) AlMgSi1-O; and (**d**) AlCu5-O AMSFs. The lines and direction of measurements are shown in the inset images.

These reactions can be beneficial because a strong bonding layer can be formed on the surface of the hollow spheres. However, if the system is not cooled fast enough the reactions can result in the complete dissolution of the hollow spheres’ walls and the composite may lose its foam structure. To investigate the interface layer energy dispersive spectroscopy (EDS) measurements along lines perpendicular to the wall of a hollow sphere were performed as shown in Figure 2. The measurements were always started from the matrix material in the direction of the hollow spheres.

In the simplest case (Al99.5-O AMSFs), an approximately 5-μm thick interface layer can be observed between the hollow sphere and the matrix material (Figure 2a). At the middle of the interface layer, an O peak can be observed because the Fe hollow spheres were exposed a slight oxidation during the preheating process of the production (see Section 3.1). There were no needle-like precipitations (common for Al-Fe intermetallics) observed in the vicinity of the outer surface. In the case of AlSi12-O foams (Figure 2b), the thickness of the interface layer was ~10 μm. Near to the outer edge (left side) of the wall a primer Si precipitation (Si peak in the graph) can be observed. The Fe content increased rapidly from ~7.5 μm, indicating the outer edge of the wall. In the next ~10 μm, the Al and O content also increased indicating the reaction between the oxide layer on the outer surface of the spheres and liquid aluminum, presumably resulting in a thin Al_2_O_3_ layer. Figure 2c shows a more complex situation in which the iron hollow sphere was originally broken and the liquid aluminum could fulfill the sphere during the production (infiltration). Higher O content was measured at both the outer and inner surfaces of the sphere due to the oxidization during the pre-heating. The thickness of the interface layers measured ~10 μm. Again, the O had peaks at both surfaces along with the stabilization of Al content confirming the afore-mentioned reaction between Al and O. The sudden changes in the Fe content and parallel in the Al and O content highlight the porous nature of the iron spheres’ wall; these porosities were also penetrated by Al in the case of this distinguished broken sphere. A similar, infiltrated sphere from the AlCu5-O foam is shown in Figure 2d. Besides the oxide layer on the wall surfaces and the corresponding changes in the Al content, a CuAl_2_ precipitation was crossed just before the outer surface of the sphere. Additionally, some Cu was also detected in the ~10 μm thick interface layers along with the Al and O content, indicating that, during cooling, the Cu rich particles tend to solidify on the surface of the spheres.

**Figure 3:**
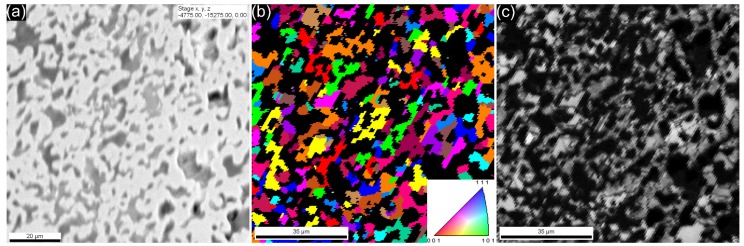
(**a**) SEM image; (**b**) EBSD image; and (**c**) image quality map of the iron sphere wall (cross-section).

The EBSD investigation of the cross-section of the iron hollow sphere walls indicated polycrystalline structure and quite extensive porosity. The porosity can be observed in Figure 3a in the form of dark grey or black areas. Figure 3b shows the inverse pole image of the investigated area, no distinguished crystalline directions were observed, the black areas are representing the porosity from which no signs could be gathered. Figure 3c represents the image quality map of the investigated area, showing acceptable sign strength: the average image quality was 135 (image quality is a measure of the Kikuchi bands’ intensities, which is linked with the sharpness of the bands; higher image quality means more reliable results). For a porous and soft material, like the wall of the spheres, 135 can be considered as satisfying image quality.

**Figure 4:**
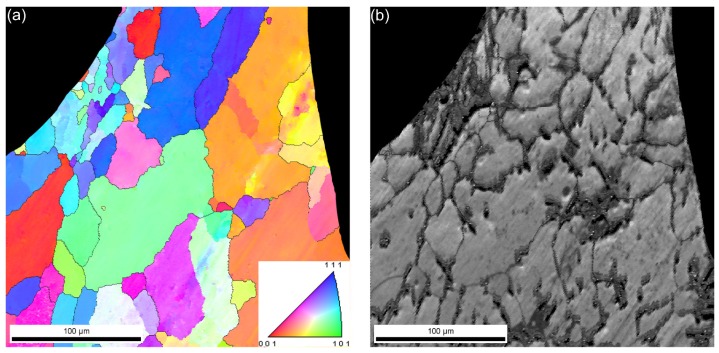
(**a**) EBSD image and (**b**) image quality map of Al99.5-O AMSF.

EBSD investigations were also performed close to the hollow spheres in order to explore any effect on the grain size or grain alignment (a typical site is shown in Figure 4). In the very vicinity of the hollow spheres, the average grain size in the matrix material was somewhat lower than far from the spheres. This can be explained by the effect of the roughness of hollow spheres on the solidification mechanism, as grain initialization site. Both the larger and smaller grains are equiaxed. Regarding the alignment and orientation of the individual grains, no distinguished directions have been found. The image quality map shows good sign strength from the larger grains, but the smaller ones resulted in lower image quality.

### 2.2. Compressive Behavior and Properties

**Figure 5:**
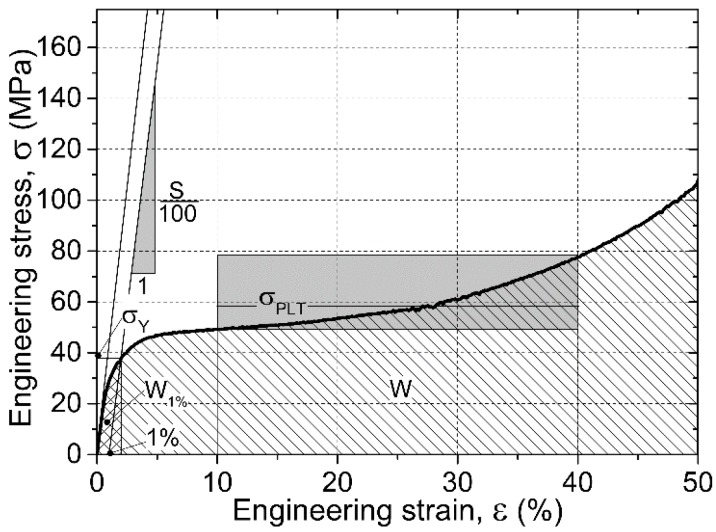
Typical compressive response of AMSFs and the interpretation of the characteristic properties.

During the compression tests, the engineering stress–engineering strain curves from the measurements were registered. A typical curve is shown in Figure 5. The recorded graphs follow the typical form of metallic foams, with a short linearly elastic section, a plateau region and subsequent densification. There are some characteristic properties that can be obtained from the curves according to the ruling standard [33]. The most important characteristic property in the design point of view is the yield strength (${\text{σ}}_{Y}$ (MPa)) that was determined at 1% plastic deformation. At this strain and corresponding stress level, macroscopic cracks could be observed on the surface of the specimens; the cracks usually initiated in the vicinity of the hollow spheres. The next important strength value is the plateau strength ${\text{σ}}_{PLT}$ (MPa)), that was determined as the average stress level between 10% and 40% strains (the limits were selected according to the recommendations of the standard [33]). The investigated AMSFs showed continuously increasing plateau region representing deformation hardening nature. Besides the strength values the structural stiffness S (MPa)) of the material (recognized as the slope of the initial part of the graph) is also an important (elastic) property. Finally, the energy values absorbed up to the appearance of the first crack and up to the end of the compression are also important characteristic properties, respectively. The energy level required to initialize the fracture process can be calculated as the integral of the curve up to 1% overall deformation (the area under the curve up to 1%, $W_{1\%}$ (MJ·m^−3^)). The overall absorbed mechanical energy W (MJ·m^−3^) can be determined as the area under the whole curve. The aforementioned mechanical properties are the characteristic properties of the AMSFs and they were monitored in the case of all produced material types. Figure 5 shows the graphical presentation of the characteristic properties.

**Figure 6:**
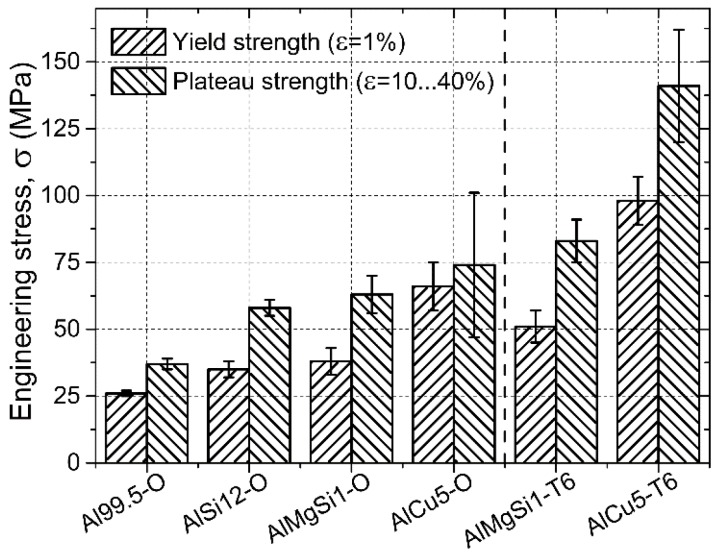
Yield and plateau strength values of the investigated AMSFs.

Regarding the strength values (Figure 6), the Al99.5-O AMSFs showed the lowest yield and plateau strengths. Both characteristic strengths increased significantly due to the alloying (solution hardening), however the alloying elements had quite different efficiency: Cu was found to be the most effective and resulted in ~+40 MPa (~150%) and ~+35 MPa (~100%) higher yield and plateau strength compared to Al99.5-O specimens, respectively. Note that the higher strength AlCu5-O AMSFs had significantly wider standard deviation because of the more stochastic fracture behavior of the higher strength matrix material. The T6 heat-treated specimens showed even higher yield strength values, generally ~30% higher than the specimens with solution heat treatment. Compared to the Al99.5-O samples (${\text{σ}}_{Y}$ = 26 ± 1 MPa) about four times higher yield strength can be achieved by T6 treated AlCu5 matrix (${\text{σ}}_{Y}$ = 98 ± 9 MPa). Moreover, the aged specimens also had higher plateau strength values than that of the solution heat-treated ones, as it was expected. The plateau strength is also important in the energy absorption point of view. The stress level of this region has distinguished role. For example, in the case of collision dampers, longer plateaus characterized by lower stress levels are more beneficial than the shorter plateaus with higher stress levels, because the reaction forces and the effects on the personnel may be lower.

**Figure 7:**
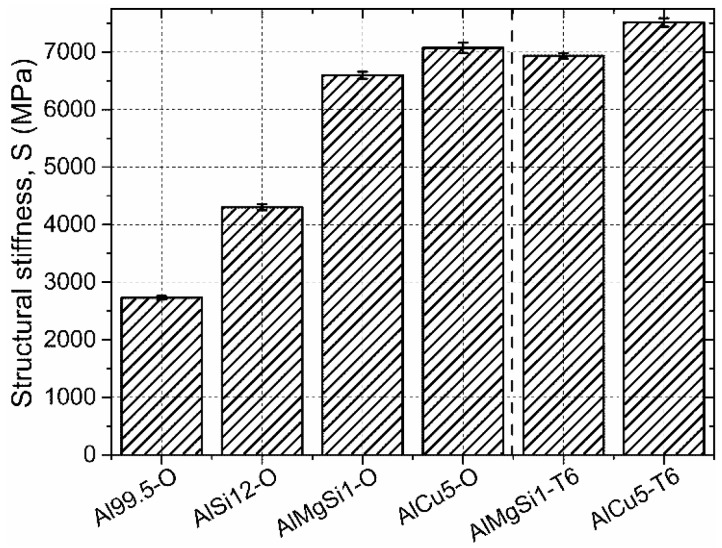
Structural stiffness values of the investigated AMSFs.

Considering the structural stiffness values (Figure 7), again, the unalloyed matrix AMSF showed the lowest value. Both the low amount Mg-Si and Cu alloying exhibited significant improvement, similarly to the quite large amount of Si alloying in the case of AlSi12-O specimens. The latter, nearly eutectic matrix had about twice as high stiffness, than the unalloyed Al99.5 samples, due to the high Young modulus of the Si precipitations (130–169 MPa, depending on the crystal orientation). In T6 treated conditions, the hardening mechanism increased the structural stiffness as well, the increment was minimal, ~5%–10% as it was experienced in the case of strength values too.

**Figure 8:**
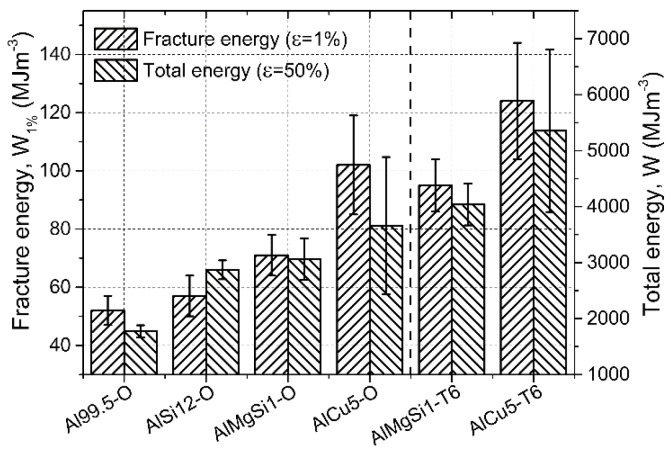
Absorbed mechanical energy values of the investigated AMSFs up to the initial fracture (ε = 1%) and to the end of the test (ε = 50%).

The fracture energies followed the trends of the yield strength (Figure 8), because the limits of the integration aiming to calculate the fracture energies were the same. The highest fracture energy therefore was shown by the AlCu5-O foams. The same trends were observed in the case of the total absorbed energies: the stochastic nature and higher scatter [34] of the plateau and densification region was more pronounced, as it can be also deduced from the higher scatter bands plotted around the discrete values in Figure 8. The T6 heat treatment ensured higher absorbed energies in the case of AlMgSi1-O and AlCu5-O AMSFs, respectively. The increment in the absorbed energies was ~30% in both cases of fracture and total energies.

As it could be seen in the previous paragraphs the mechanical properties of the investigated AMSFs are outstanding compared to the conventional metallic foams and therefore they are promising materials for collision dampers and lightweight structural parts.

## 3. Experimental Section

In this section, the production process, the applied materials, and the circumstances and specialties of the investigations and sample preparation are detailed.

### 3.1. Production of the AMSF Blocks and Samples

Four different AMSF were produced by low-pressure inert gas assisted infiltration technique. The applied matrices were Al alloys (Table 1). Globomet (GM) grade hollow pure Fe spheres were used as filler material (supplied by Hollomet GmbH, Dresden, Germany [35]). The average diameter of the hollow spheres was 1.92 ± 0.07 mm (obtained by measuring 1000 hollow spheres on an Olympus SZX 16 stereo microscope). The nominal wall thickness of the hollow spheres was 23 ± 0.6 μm, while their density was 0.393 g·cm^−3^.

**Table 1:** Chemical composition and basic properties of the constituent materials (measured by EDS).

Matrix	Chemical Element (wt %)						Young Modulus, *E*_0_ (GPa)	Density, *ρ* (g·cm^−3^)
	Al	Mg	Si	Cu	Fe	Other		
Al99.5	99.5	–	0.1	–	0.1	0.3	69.0	2.71
AlSi12	86.0	0.1	12.8	–	0.1	1.0	78.6	2.65
AlMgSi1	97.0	1.1	1.1	–	0.5	0.3	70.0	2.70
AlCu5	95.0	–	–	4.5	–	0.5	73.1	2.81
Fe sphere wall	–	–	–	–	99.9	0.1	212.0	7.80

**Figure 9:**
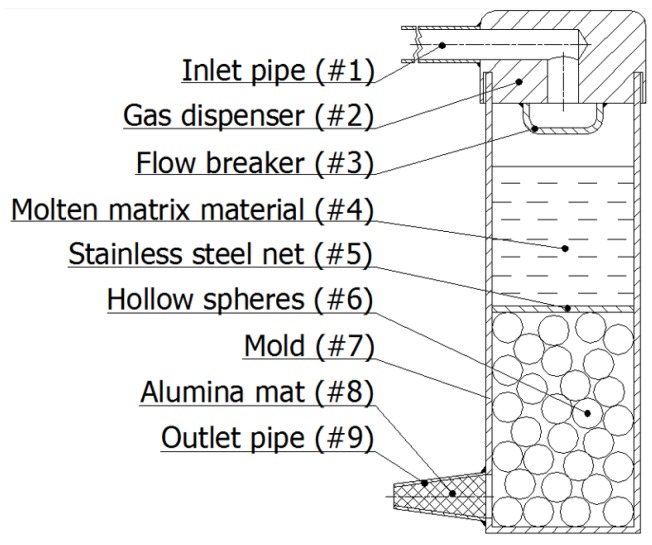
Schematic sketch of the infiltration chamber.

For the infiltration process, a special mold was prepared (Figure 9). The mold (#7) was coated by a FormKote T-50 graphite layer (Everlube Products, Peachtree City, GA) in order to facilitate the composite removal. The mold was filled halfway by the hollow spheres (#6) during continuous tapping (to achieve ~65% volume fraction [36,37]). The filler was fixed in position by a 316L stainless steel net (#5) and pre-heated in a furnace (Lindberg/Blue M) to 300 °C for 0.5 h. Meanwhile, the matrix material was melted and overheated (T_melting_ + 50 °C) in a Power-Trak 15–96 induction furnace. The molten matrix material (#4) was poured into the mold and the inert gas (Ar) was injected into the system through a pressure reducer and the pipe system (#1 and #2) to ensure the 400 kPa infiltration pressure. The gas from the spaces between the hollow spheres was exhausted through an Al_2_O_3_ mat (#9) stuffed pipe (#8) at the bottom of the mold. After rapid solidification, the mold was opened by milling and the AMSF block was removed. For further details, please refer to [34].

The density of the AMSF blocks were measured by Archimedes’ method: 1.41 g·cm^−3^, 1.42 g·cm^−3^, 1.60 g·cm^−3^ and 1.72 g·cm^−3^ for the Al99.5, AlSi12, AlMgSi1 and AlCu5 AMSFs, respectively. The AMSF blocks were solution treated (at 520 °C for 1 h, water cooled). Due to the cold aging nature of the Cu containing Al alloys, the tests were done immediately after the cooling to avoid any effect of natural aging. The AlMgSi1 and AlCu5 specimens were also tested in aged condition (T6 treatment: (i) homogenization at 530 °C for 1 h, water cooled and (ii) aging at 170 °C for 14 h, water cooled). Again, the specimens were tested immediately after the aging.

### 3.2. Sample Preparation and Microstructural Analysis

The samples for the microscopic investigations were cold mounted and prepared according to the details of Table 2. The grinding and polishing steps were performed on a Buehler Beta automatic grinding-polishing machine. The optical microscopy images were taken on an Olympus PMG3 microscope. For the optical microscopic images, the samples were etched by Keller’s reagent (95 mL H_2_O, 2.5 mL HNO_3_, 1.5 mL HCl and 1 mL HF). The SEM images and EDS investigations were performed on a Philips XL 30 electron microscope with EDAX Genesis EDS and TSL EBSD detector. EDS line analyses were done on polished surfaces. The acceleration voltage was 20 kV. The measurement started from the matrix materials and crossed the wall of the hollow sphere. Each point was excited for 30 s with 35 μs amplification time. The EBSD measurements required special sample preparation. In the case of the iron hollow sphere walls, the method in Table 2 was extended by an additional step: polishing on Buehler Vibromet 2 (microcloth, 0.05 SiO suspension, 610 g load). In the case of the matrix material, electro-polishing (Jean Wirtz Poliomat) was applied after the standard preparation (Table 2). The applied voltage was 100 V, the polishing time was 30 s. The electrolyte consisted of 840 mL ethanol, 125 mL glycerin and 35 mL perchlorethylene acid (65% concentration). For the EBSD investigations, the samples were tilted by 70°, the acceleration voltage was 25 kV and the investigated area covered 30,000 measurement points.

**Table 2:** The steps of the applied grinding and polishing process.

Abrasive	Time (min)	Load (N)	Revolution (min^−1^)	Direction
P 320 SiC	1	22	220	counter
P 600 SiC	1	22	220	counter
P 1200 SiC	1	22	220	counter
P 2400 SiC	1	22	220	counter
6 μm diamond	15	27	150	counter
3 μm diamond	6	27	150	counter
0.05 μm SiO	3	27	125	comply

### 3.3. Mechanical Tests

Six cylindrical specimens for compression tests were machined from each block. The specimens were designated according to their matrix and heat treatment (for example, Al99.5-O stands for a specimen with Al99.5 matrix and ~65 vol % GM grade hollow spheres in solution treated condition). The diameter (D) and the height (H) of the specimens were 14 mm (H/D = 1). The compression tests were done on a MTS 810 type universal testing machine in a four column tool at room temperature. The surfaces of the tool were hardened to 45 HRC, ground and polished. The specimens and the tool were lubricated with MoS_2_ content anti-seize material. The strain rate was 0.01 s^−1^ (quasi-static condition). The engineering stress–engineering strain curves were registered and processed according to the standard for the compression tests of cellular materials (DIN50134:2008 [33]).

## 4. Conclusions

From the detailed investigations discussed above, the following conclusions can be drawn.

- Low-pressure inert gas infiltration is a proper method to produce AMSFs with high volume fraction of hollow sphere inclusions.
- Depending on the matrix material, a thin interface layer may be formed between the hollow spheres and the matrix. This layer ensures good bonding and load transfer, resulting in favorable mechanical properties. EBSD investigations showed that the average grain size in the vicinity of the hollow spheres was lower than in the matrix, far from the hollow spheres. The grains had no distinguished directions and were equiaxed.
- The standardized mechanical tests revealed beneficial specific mechanical properties. The matrix material had significant effect on the mechanical properties, as well as the T6 heat treatment (solution treated and artificially aged). The yield strength (at 1% deformation), the plateau strength (between 10% and 40% deformation) and the energy absorption capabilities up to the initialization of fracture (1% deformation) and up to the end of the test (50% deformation) were increased by ~30% in the case of T6 treatment. The structural stiffness was also varied with the matrix material, but remained almost unchanged in the case of T6 treatment.
